# Genome-wide linkage analyses of two repetitive behavior phenotypes in Utah pedigrees with autism spectrum disorders

**DOI:** 10.1186/2040-2392-1-3

**Published:** 2010-02-22

**Authors:** Dale S Cannon, Judith S Miller, Reid J Robison, Michele E Villalobos, Natalie K Wahmhoff, Kristina Allen-Brady, William M McMahon, Hilary Coon

**Affiliations:** 1 Utah Autism Research Project, Department of Psychiatry, University of Utah, 650 Komas Drive, Suite 206, Salt Lake City, UT, 84108-3528, USA

## Abstract

**Background:**

It has been suggested that efforts to identify genetic risk markers of autism spectrum disorder (ASD) would benefit from the analysis of more narrowly defined ASD phenotypes. Previous research indicates that 'insistence on sameness' (IS) and 'repetitive sensory-motor actions' (RSMA) are two factors within the ASD 'repetitive and stereotyped behavior' domain. The primary aim of this study was to identify genetic risk markers of both factors to allow comparison of those markers with one another and with markers found in the same set of pedigrees using ASD diagnosis as the phenotype. Thus, we empirically addresses the possibilities that more narrowly defined phenotypes improve linkage analysis signals and that different narrowly defined phenotypes are associated with different loci. Secondary aims were to examine the correlates of IS and RSMA and to assess the heritability of both scales.

**Methods:**

A genome-wide linkage analysis was conducted with a sample of 70 multiplex ASD pedigrees using IS and RSMA as phenotypes. Genotyping services were provided by the Center for Inherited Disease Research using the 6 K single nucleotide polymorphism linkage panel. Analysis was done using the multipoint linkage software program MCLINK, a Markov chain Monte Carlo (MCMC) method that allows for multilocus linkage analysis on large extended pedigrees.

**Results:**

Genome-wide significance was observed for IS at 2q37.1-q37.3 (dominant model heterogeneity lod score (hlod) 3.42) and for RSMA at 15q13.1-q14 (recessive model hlod 3.93). We found some linkage signals that overlapped and others that were not observed in our previous linkage analysis of the ASD phenotype in the same pedigrees, and regions varied in the range of phenotypes with which they were linked. A new finding with respect to IS was that it is positively associated with IQ if the IS-RSMA correlation is statistically controlled.

**Conclusions:**

The finding that IS and RSMA are linked to different regions that only partially overlap regions previously identified with ASD as the phenotype supports the value of including multiple, narrowly defined phenotypes in ASD genetic research. Further, we replicated previous reports indicating that RSMA is more strongly associated than IS with measures of ASD severity.

## Background

Although it is generally accepted that genetic factors play a major role in the etiology of autism spectrum disorders (ASDs)[1], identification of specific genetic risk markers is complicated by the phenotypic complexity of clinical diagnoses. For example, the *Diagnostic and Statistical Manual of Mental Disorders 4*^*th*^*ed. *(DSM-IV)[2] diagnostic criteria for autistic disorder (AD) require impairments in three domains: social interaction, communication and repetitive and stereotyped behavior. Each of these three domains has been shown to be heritable, but their covariation in the general population is modest, and genetic modeling suggests distinct genetic influences for each [3-5]. Thus, it has been argued that the ability to identify susceptibility loci for ASD would be increased if specific ASD/AD traits were used as phenotypes [3,6].

Specific ASD/AD traits have been employed in genetic studies most often either to stratify pedigrees for linkage analysis or as the dependent variable in association tests for specific alleles. For example, the first approach has found stronger ASD linkage signals in pedigrees with more abnormal levels of phrased speech delay [7,8], repetitive behavior [9-11] and savant skills [12], but there have been failures in replication [13]. The second approach has resulted in significant genotype associations with repetitive behavior [14-16]. A third, less common approach has been to use the specific trait as a quantitative or qualitative phenotype in linkage analyses. For example, we used the Social Reciprocity Responsiveness Scale (SRS) [17] score as the phenotype in linkage analyses of multiplex ASD pedigrees (Coon *et al*., Genome-wide linkage using the Social Responsiveness Scale (SRS) in Utah autism pedigrees, submitted). Although each of these methods has merit, it should be noted that the first method attempts to reduce heterogeneity of the diagnostic phenotype by stratification on a specific trait, whereas the second and third approaches seek to identify risk markers for the trait itself.

Repetitive and stereotyped behavior is a promising candidate for further genetic study because it probably comprises at least two even more specific phenotypes that differ in their behavioral correlates, familiality, and relation to genetic linkage with ASD. The 'restricted and repetitive stereotyped behavior' (RRSB) domain of the Autism Diagnostic Interview--Revised (ADI-R) [18,19] is a well-accepted measure of the repetitive behavior phenotype. To uncover the factor structure of RRSB, a variety of factor analytic techniques have been used with different subsets of RRSB items and with study populations that differ in ASD severity and ethnicity [11,20-25]. Remarkably, in spite of their methodological differences, these analyses converge on a two-factor solution comprising 'repetitive sensory-motor actions' (RSMA) and 'insistence on sameness' (IS). RSMA items investigate repetitive physical mannerisms and unusual sensory interests, whereas IS items investigate compulsive behaviors. There are two exceptions to the common two-factor solution. First, an exploratory factor analysis of RRSB items [26] recovered essentially the same RSMA and IS factors but also found a third factor ('circumscribed interests'). This finding does not detract from the conclusion that RRSB comprises RSMA and IS, but rather suggests that RRSB may measure additional factors as well. Second, a principal components analysis of all ADI-R items identified six factors, including a 'compulsions' factor that contained some items from both the IS and RSMA factors, and a 'social intent' factor that combined social interaction items with the RSMA item of 'hand and finger mannerisms' [27]. Despite this, the preponderance of statistical evidence indicates that RSMA and IS are distinct factors within the RRSB domain.

It is well established that IS and RSMA have different patterns of relationship with other ASD traits. Specifically, RSMA, but not IS, has been reported to be associated with lower IQ, less adaptive behavior, and later age of appearance of first words and phrases [6,20,21], which suggests that RSMA may be more correlated with ASD severity [6]. These findings support the validity of treating IS and RSMA as different phenotypes.

There is more empirical support for a genetic effect on IS than on RSMA. Whereas modest evidence of familial concordance occurs for IS, no reported concordance occurs for RSMA [21,25]. Thus, the IS factor may account for earlier findings that RRSB is familial [28,29]. Indeed, Silverman *et al*. [28] reported that RRSB categories that include IS items were familial, whereas those that include RSMA items were not. Further, a linkage analysis across the 15q11-q13 region in a subset of families with the highest IS scores resulted in increased LOD scores for AD [11] over scores obtained without stratification. By contrast, stratification on RRSB or RSMA did not increase lod scores. Finally, obsessive compulsive disorder (OCD) features in parents were associated with IS, but not RSMA, in children with AD [30], which suggests that IS may be part of a broader autism phenotype of obsessive behavior.

We are not aware of previous genetic linkage studies with either IS or RSMA as the phenotype. The primary aim of the present study was to perform a genome-wide linkage analysis with both IS and RSMA as phenotypes using large extended ASD pedigrees. Thus, our goal was to identify genetic risk regions for IS and RSMA in ASD cases rather than to stratify on IS and RSMA to reduce ASD heterogeneity. Because IS and RSMA data were available only for ASD cases rather than for all pedigree members, we focused our analyses on these specific phenotypes in ASD cases and did not include clinically unaffected family members in this study. Signals obtained with these phenotypes were compared with those found in the same set of pedigrees using ASD diagnosis [31]. Contrasting results obtained with IS and RSMA with those obtained by ASD categorical diagnosis addresses empirically the possibilities that more narrowly defined phenotypes improve linkage analysis signals, and that different narrowly defined phenotypes are associated with different loci. Secondary aims were to examine the correlates of IS and RSMA and to assess the heritability of both scales.

## Methods

This study has ongoing approval from the University of Utah institutional review board (IRB). All adults participating in the research signed informed consent documents. All subjects under the age of 18 signed assent documents and their parents or guardians signed parental permission documents. These documents were approved by the University of Utah IRB.

### Subjects

Subjects were members of 70 pedigrees having at least two family members with ASD. In total, 653 subjects were genotyped, 192 of whom had a study diagnosis of ASD. Study diagnosis was based in almost all instances on both the ADI-R [18,19] and the Autism Diagnostic Observation Schedule-Generic (ADOS-G) [32]. These pedigrees were used in our recent genome-wide linkage analyses of ASD [31]. All of the families studied are part of the Utah collection of multiplex ASD pedigrees. We did not include pedigrees from other collections or repositories. Additional sample characteristics including pedigree sizes, ascertainment and assessment methods were reported previously [31].

### Phenotypes

#### RSMA and IS scales

RSMA and IS scales were derived from the RRSB domain of the ADI-R, which was available for 183 subjects with a study diagnosis of ASD. RSMA and IS items were ADI-R items that reliably loaded on one scale or the other in previous factor analytic studies [11,20-25]. For both scales, scores were the unweighted sum of ADI-R item 'ever' ratings of 0-3. We believe this method of scoring the two scales is less susceptible to chance inter-item correlations in our data than would be factor scales derived from our data alone. RSMA items included 'hand and finger mannerisms', 'unusual sensory interests', 'repetitive use of objects', 'complex mannerisms' and 'rocking'. IS items included 'difficulties with minor changes in personal routine or environment', 'resistance to trivial changes in environment' and 'compulsions/rituals'.

#### Language delay

Items from the ADI-R ('age of first words' and 'age of first phrases') were used to assess language delay in ASD cases. For parents who indicated normal onset but who could not remember the exact ages, values were set to 23 months for words and 32 months for phrases (acquiring language after these ages is considered abnormal on the ADI-R). For parents who indicated delayed onset but could not remember the exact ages, values were set to 1.5 standard deviations above the mean. For subjects who never acquired language, values were set to 3 standard deviations above the mean.

#### Intellectual function

IQ was measured in subjects with ASD using an assessment instrument appropriate for the subject's age and developmental level. IQ measures included the Wechsler Intelligence Scale for Children, 3rd revision (WISC-III) [33], the Wechsler Adult Intelligence Scale, 3rd revision (WAIS-III) [34], the Differential Abilities Scale (DAS) [35] and the Mullen Scales of Early Development [36].

#### SRS

The SRS is a quantitative measure of social ability ranging continuously from significantly impaired to above-average social abilities [17]. Although the SRS can be used with a general population, in our study the SRS was used only with ASD cases. The SRS mannerisms scale, which contains items that measure stereotypical behaviors and restricted interests, was used to determine whether IS or RSMA was more highly associated with another accepted measure of repetitive behavior in ASD cases.

### Genotyping

Genotyping services were provided by the Center for Inherited Disease Research (CIDR), using the 6 K single nucleotide polymorphism (SNP) linkage panel. Methods and quality control procedures have been described in detail previously [31]. After quality control, there were genotypes from 6,044 SNPs on 653 pedigree members who were members of 67 informative families. Eliminating linkage disequilibrium (LD) between markers in linkage studies has been strongly recommended, as false-positive results can occur in the presence of LD, particularly with extended multigenerational pedigrees for which ancestral genotypes are unavailable [37]. Recommended thresholds of acceptable LD vary, but a pair-wise r^2 ^value of 0.05 between SNPs has been supported with extensive simulation studies [37]. Therefore, before linkage analysis, we screened SNPs for LD using the PLINK software package [38], which recursively removes SNPs within a sliding window. We set a window size of 50 SNPs, shifted the window by 5 SNPs at each step, and used a variance inflation factor (VIF) of 1.5, which is equivalent to an r^2 ^of 0.33 regressed simultaneously over all SNPs in the selected window. This r^2 ^considers not only the correlations between SNPs but also between linear combinations of SNPs [38], and corresponds in our data to a pair-wise r^2 ^value of approximately 0.05. This screening for LD removed 1,207 SNPs. As part of the validation procedure, we also removed 115 SNPs with a minor allele frequency < 0.10 and 4 SNPs that were not in Hardy-Weinberg equilibrium (standard 1 degree of freedom test failed at the 0.05 level). The total number of SNPs left after this phase was 4,718.

### Analyses

#### Heritability

The heritability (proportion of variance in the trait due to genetic influences) of IS and RSMA was estimated using SOLAR software [39]. For discrete traits, SOLAR uses a threshold model to estimate polygenic heritability [40]. Estimates were also computed using jPAP software [41]; no substantive differences were found.

#### Linkage analysis

We used the genetic map provided by CIDR based on the deCODE genetic map [42]. Base pair positions were obtained from the March 2006 human reference sequence (hg18) assembly. Analysis was performed using the multipoint linkage software MCLINK, a Markov chain Monte Carlo (MCMC) method that allows for multilocus linkage analysis on large extended pedigrees [43]. Using blocked Gibbs sampling, MCLINK generates inheritance vectors from the Markov chain. Each state in this chain is an inheritance state, indicating the grandpaternal or grandmaternal origin of an allele at each marker locus, with changes in the origin of alleles along the inheritance vector indicating points of recombination. MCLINK then estimates the log-likelihood function linkage statistics. Internally, MCLINK runs the analysis five times to ensure a consistent solution. MCLINK has been used previously to identify candidate genomic regions for a number of complex diseases [44-48]. Results from MCLINK have shown a high degree of similarity to other MCMC linkage methods [49], and to exact linkage methods and variance components linkage methods as applied to extended pedigrees [50]. Allele frequencies for the MCLINK analysis were estimated using all of the observed data.

We performed nonparametric and general parametric model-based analyses. Although nonparametric methods are the standard analytic approach for complex psychiatric disorders, parametric methods have some advantages in the analysis of a complex trait such as ASD, particularly when using large extended pedigrees. Parametric models, which are based on assumptions about the genotype-phenotype relationship, simplify the parameter space and allow for more powerful and efficient analyses without leading to false-positive results [51,52]. We decided to use two simple dominant and recessive models based on an extensive set of simulation analyses in which the results of various simple inheritance models were compared with the results of analyses based on a specified true model of inheritance [53]. Those simulation analyses found that the power to reach a given lod score using the simple models was approximately 80% that of the true model, and that the expected lod scores for the simple models approached the true expected lod scores. The multipoint hlod score allows for unlinked pedigrees and variation in the recombination fraction. The HLOD provided by MCLINK is robust to model mis-specification, and may reflect the true position of linkage regions more accurately under conditions of appreciable heterogeneity [54]. HLOD scores have been shown to be more powerful than homogeneity LOD scores or model-free methods under these conditions [55,56]. The HLOD has been shown to produce scores consistent with other published methods [57,58].

For both IS and RSMA, the phenotype was coded as unknown if the measure was not available, unaffected if the score was in the lowest tertile for the scale, and affected if the score was in the upper two tertiles. This approach re-codes affection status for all subjects rather than selecting a subset of subjects with high values on the traits. For IS, raw score tertile bins were 0-1, 2-3 and > 3; for RSMA, they were 0-3, 4-6 and > 6. The tertiles were given different liability classes (penetrances) to weight those in the upper tertile more strongly. Our recessive model assumed a disease allele frequency of 0.05 and penetrances of each of the three genotypes of 0.0014, 0.0014 and 0.8 in the upper tertile, and 0.01, 0.01 and 0.5 in the middle tertile. For the dominant model, the disease allele frequency was 0.0025. The penetrances were 0.0014, 0.8 and 0.8 in the upper tertile, and 0.01, 0.5 and 0.5 in the middle tertile. These model parameters roughly reproduce the reported population frequency of ASDs [1].

Linkage analyses were repeated on the basis of residual scale scores to determine whether signals could be replicated using measures of IS and RSMA phenotypes that were statistically independent of each scale's correlation with the other. Thus, for each scale, residual scores were computed using the other scale as a covariate (that is, IS-Adj = IS adjusted for RSMA and RSMA-Adj = RSMA adjusted for IS). Then, residual scores were divided into tertiles, and phenotype and liability values were coded in the same manner as were raw scores, that is, the lowest tertile was coded as unaffected and the top two tertiles were coded as affected, and the penetrance of the highest tertile was greater than that of the lower two tertiles.

For HLOD scores, results are presented using the Lander and Kruglyak [59] genome-wide criteria. Suggestive linkage evidence was defined by a LOD score ≥ 1.86 and significant genome-wide linkage evidence was defined by a LOD score ≥ 3.30.

## Results

### Interscale correlation

The zero-order correlation between RSMA and IS was r = 0.32 (*P *< 0.001), indicating that they share 10% of their variance (r^2 ^= 0.32^2 ^= 0.10). Consequently, residual scores were closely correlated with the raw score of the same scale (correlations = 0.95, P-values < 0.001), and 90% of the variance of each scale was unique (r^2 ^= 0.95^2 ^= 0.90).

### Scale correlates

RSMA was more strongly associated than IS with other ASD features (Table 1). Both IS and RSMA raw scores were correlated with ADI-R domain scores and SRS mannerisms scale, but the RSMA correlations with ADI-R social and SRS mannerisms scales were significantly greater than those for IS. RSMA but not IS was correlated with ADOS score (after controlling for the effect of ADOS module scale), age of first phrases and IQ measures. With the exception of IQ measures, criterion variables significantly associated with raw scale scores tended to have lower correlations with residual scores, which suggests that the variance that IS and RSMA have in common may reflect a broader ASD trait. IQ measures, which were negatively correlated with RSMA, tended to be even more negatively associated with RSMA-Adj, although this trend was nominally significant (*P *< 0.01) only for non-verbal IQ. IS-Adj was positively correlated with IQ measures even though raw score IS was not, and IS-IQ correlations were significantly higher with residual than with raw scores. Thus, the unique variance of both IS and RSMA was less strongly associated with ASD but more strongly associated with IQ, although the direction of the relations with IQ was opposite (Table 1).

**Table 1:** Correlations of IS and RSMA with ADI-R, ADOS, SRS and IQ measures.

Criterion	IS		RSMA		t-Test		
	Raw	Res	Raw	Res	IS vs. RSMA	IS vs. IS-Adj	RSMA vs. RSMA-Adj
ADI-R Social	0.30*	0.12	0.57*	0.50*	3.75*	8.98*	3.62*
ADI-R Comm	0.41*	0.27*	0.49*	0.40*	0.98	6.09*	4.19*
ADI-R RRSB	0.63*	0.46*	0.61*	0.42*	0.46	10.95*	11.77*
ADOS Score†	- 0.01	- 0.10	0.29*	0.31*	3.60*	3.88*	0.87
SRS Mannerisms	0.29*	0.11	0.57*	0.51*	3.70*	8.60*	3.03*
First words	-0.02	- 0.06	0.12	0.14	1.43	1.51	0.52
First phrases	0.10	0.03	0.22*	0.21*	1.18	2.26	0.36
VIQ	0.10	0.23*	- 0.38*	- 0.43*	5.52*	5.48*	2.09
NVIQ	0.17	0.30*	- 0.37*	- 0.45*	6.42*	5.68*	3.31*
FSIQ	0.11	0.25*	- 0.41*	- 0.47*	6.26*	6.25*	2.56

ADI-R Comm, verbal communication; ADOS, Autism Diagnostic Observation Schedule; FSIQ, full scale IQ; IS, insistence on sameness; IS-Adj, IS adjusted for RSMA; NVIQ, nonverbal IQ; RRSB, restricted and repetitive stereotyped behavior; RSMA, repetitive sensory-motor actions; RSMA-Adj, RSMA adjusted for IS; SRS, Social Reciprocity Scale; VIQ, verbal IQ.

*P < 0.01.

†For ADOS score, partial correlations with ADOS 'module' effects removed are reported.

The t-tests are two-tailed tests of the difference between correlations with the same criterion variable [70]. Sample sizes ranged from 131 to181, depending criterion variable.

### Heritability

The heritability of both scales was significant. For IS, H^2 ^was 0.85 (*P *< 0.0004, SE = 0.21), and for RSMA, H^2 ^was 0.51 (*P *< 0.03, SE = 0.26). Because the scales were significantly correlated, we also estimated the heritability of each with the other as a covariate. With RSMA as a covariate, IS was still significant (H^2 ^= 0.69, *P *< 0.004, SE = 0.23) and RSMA was a significant covariate (*P *= 0.003). By contrast, when IS was entered as a covariate for RSMA, RSMA was not significantly heritable (H^2 ^= 0.31, *P *= 0.13, SE = 0.27), but IS was a significant covariate (*P *< 0.0001).

### Linkage

**Figure 1:**
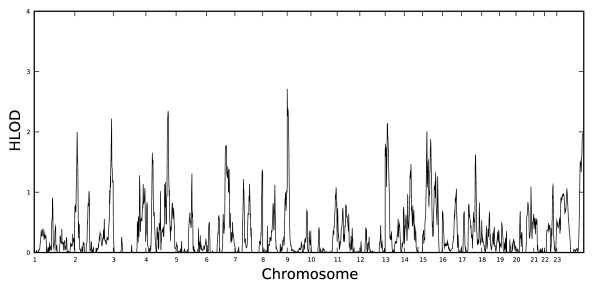
**Genome-wide distribution of recessive model HLOD scores for insistence on sameness (IS)**.

**Figure 2:**
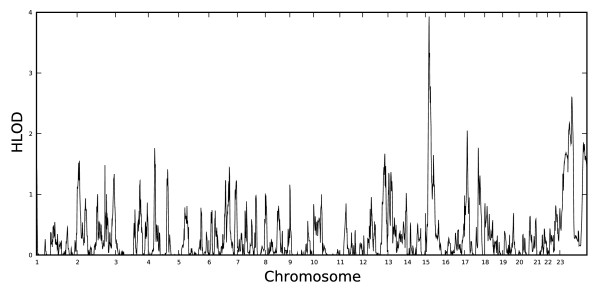
**Genome-wide distribution of recessive model HLOD scores for repetitive sensory-motor actions (RSMA)**.

Table 2 lists all regions with at least suggestive evidence of linkage (HLOD ≥ 1.86 for parametric tests [59] or *P *< 0.005 for nonparametric tests). There was strong correspondence between regions for which there was evidence of linkage with the recessive model and nonparametric linkage (NPL), which suggests that these linkage findings are resistant to model mis-specification. Fewer tests of the dominant model, compared with the recessive model, were suggestive or significant. Thus, to simplify presentation of genome-wide results, Figures 1 and 2 display the genome-wide distribution of HLOD scores for the recessive model only (Table 2, Figures 1 and 2).

**Table 2:** Linkage signals for insistence on sameness (IS) and repetitive sensory-motor actions (RSMA).

Chromosome	Marker	Boundary (Mb)	Phenotype	Rec	Dom	NPL
2p25.3-p25.1	rs309276	4.11 to 8.13	IS-Adj	2.12	-	2.56
2p25.2-p25.1	rs1560382	4.58 to 9.97	IS	2.00	-	2.56
2q37.1-q37.3	rs1569125	230.89 to 241.89	RSMA	--	2.15	-
2q37.1-q37.3	rs1198823	235.61 to 239.02	IS-Adj	--	2.02	2.57
2q37.1-q37.3	rs1198823	235.61 to 240.61	IS	2.22	3.42**	2.99
3q13.31-q22.1	rs13975	115.07 to 133.09	RSMA-Adj	--	2.02	-
4q31.22-q32.2	rs538317	146.67 to 162.95	IS-Adj	2.39	-	2.91
4q31.23-q32.2	rs2090870	150.39 to 163.91	IS	2.35	-	-
6q22.31-q24.3	rs1041480	125.57 to 148.01	IS	--	-	2.69
8q13.2-q22.1	rs1025908	68.59 to 97.25	RSMA-Adj	--	1.93	-
8q13.2-q22.1	rs2016354	70.19 to 96.31	RSMA	--	2.34	-
9p24.3-p24.1	rs1532309	0.59 to 4.80	IS	2.71	-	-
13q12.12-q12.3*	rs306395	22.86 to 30.07	IS	2.15	-	2.76
15q13.1-q14†	rs904951	27.94 to 31.72	RSMA	**3.93**	2.68	**4.54**
15q13.1-q14	rs904951	27.94 to 31.72	RSMA-Adj	**4.35**	2.19	**4.11**
15q13.1-q15.1	rs965471	27.94 to 39.04	IS-Adj	2.05	-	-
15q13.3-q15.1	rs965471	29.46 to 38.23	IS	2.00	-	-
15q21.1-q22.2	rs11856	43.47 to 60.20	IS	1.88	-	2.60
15q21.2-q22.2	rs11856	50.79 to 59.13	IS-Adj	3.03	-	3.10
17p13.2-p13.1	rs1848550	5.23 to 9.00	RSMA	2.05	-	-
17q23.2-q24.2	rs1874087	58.03 to 65.40	RSMA-Adj	2.40	-	-
22q13.1-q13.33	rs132817	37.83 to 48.44	RSMA-Adj	--	1.98	-
Xp11.4-q21.33	rs763554	40.14 to 97.88	RSMA	2.61	-	-
Xq13.1-q21.33	rs763554	70.24 to 96.70	RSMA-Adj	3.07	-	-
Xq27.3-q28	rs17318101	142.53 to 154.55	RSMA	1.86	-	-
Xq27.3-q28	rs473491	144.27 to 154.55	IS	1.97	-	-

Dom, dominant; Rec, recessive; IS, insistence on sameness; IS-Adj, IS adjusted for RSMA; RSMA, repetitive sensory-motor actions; RSMA-Adj, RSMA adjusted for IS.

*The nonparametric linkage signal on chromosome 13 for IS shifted slightly downstream: 13q12.12-q13.1, marker = rs1886204, region boundaries = 22.86 to 31.54 Mb.

†The dominant model signal on chromosome 15 for RSMA shifted slightly downstream: 15q13.1-q14, marker = rs2596156, region boundaries = 25.85 to 35.33 Mb.

'Adj', scale adjusted for the other scale. Signals that are least suggestive [60] are shown for parametric models; for nonparametric linkage (NPL), signals shown are regions where *P *< 0.005. Bold font for HLOD scores indicates genome-wide significance [60] and for NPL indicates *P *< 2E-05. Signal boundaries were defined as 1 HLOD or 1 NPL drops from the peak. If linkage at a locus was observed for more than one analysis, boundaries shown are for the recessive model.

**Figure 3:**
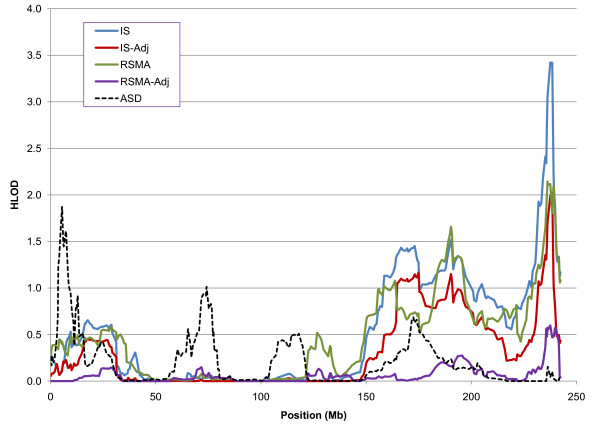
**Chromosome 2 HLOD scores for the dominant model as a function of phenotype**. IS, insistence on sameness; RSMA, repetitive sensory-motor actions; IS-Adj, IS adjusted for RSMA; RSMA-Adj, RSMA adjusted for IS. Autism spectrum disorder (ASD) HLOD scores are based on previously reported linkage analyses with the same pedigrees [31].

Evidence of linkage reached genome-wide significance levels (HLOD > 3.30) for two regions, 2q37.1-q37.3 and 15q13.1-q14 (Table 2), so we examined the linkage evidence for these regions in greater detail (Table 3). For 2q37.1-q35.3, the linkage evidence was greater for the dominant model, so dominant model HLOD scores across chromosome 2 are shown in Figure 3 along with ASD HLOD scores from our earlier work [31]. The evidence of linkage to 2q37.1-q37.3 was greater for IS than for IS-Adj, RSMA and RSMA-Adj. Note too that we observed no evidence of ASD linkage to this region in our earlier study [31]. Taken together, these findings suggest 2q37.1-q35.3 may harbor a genetic risk marker for repetitive behavior, particularly IS, which is not strongly associated with ASD (Table 3, Figure 3).

**Table 3:** HLOD values for both recessive and dominant parametric models for both unadjusted and adjusted measures of IS and RSMA for selected regions.

Region	IS		IS-Adj		RSMA		RSMA-Adj	
	Rec	Dom	Rec	Dom	Rec	Dom	Rec	Dom
2q37.1-q37.3	2.22*	3.42†	1.34	2.02*	1.33	2.15*	0.70	0.60
15q13.1-q14	2.00*	0.29	2.05*	0.91	3.93†	2.68	4.35†	2.19*
15q21.1-q22.2	1.88*	0.42	3.03*	1.02	1.65	0.94	1.02	0.92

Dom, dominant; Rec, recessive; IS, insistence on sameness; IS-Adj, IS adjusted for RSMA; RSMA, repetitive sensory-motor actions; RSMA-Adj, RSMA adjusted for IS.

*HLOD values reaching the level of suggestive evidence [60].

†HLOD values reaching genome-wide level of significance [60].

**Figure 4:**
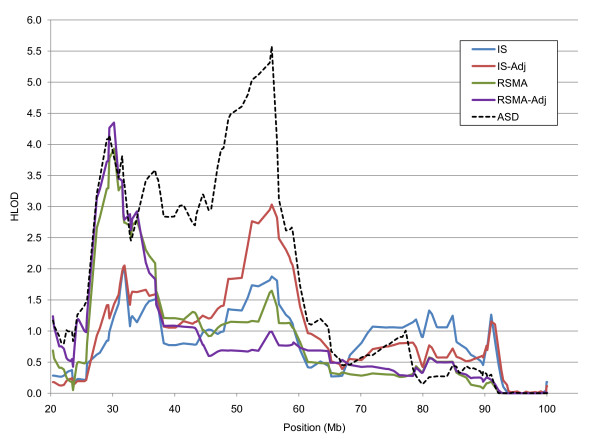
**Chromosome 15 HLOD scores for the recessive model as a function of phenotype**. IS, insistence on sameness; RSMA, repetitive sensory-motor actions; IS-Adj, IS adjusted for RSMA; RSMA-Adj, RSMA adjusted for IS. Autism spectrum disorder (ASD) HLOD scores are based on previously reported linkage analyses with the same pedigrees [31].

Linkage results for chromosome 15 were of particular interest, both because of the different pattern of signals for IS and RSMA, and the linkage magnitude. Linkage evidence for both IS and RSMA at 15q13.1-q14 was greater for the recessive than for the dominant model (Table 3). Because there also was suggestive evidence with the recessive model of IS linkage to 15q21.1-q22.2 (Table 3), Figure 4 shows HLOD scores for the recessive model across chromosome 15. The linkage evidence at 15q13.1-q14 was greater for RSMA than for IS, but nonetheless the evidence for IS was suggestive. A different pattern of findings was observed at 15q21.1-q22.2. Not only was there no RSMA signal this location, but the IS-Adj signal was much stronger than the unadjusted IS signal (HLOD = 3.03 and 1.88, respectively; NPL = 3.10 and 2.60, respectively). This was the largest difference in linkage values between adjusted and unadjusted phenotypes for any locus at which at least suggestive linkage evidence was observed for both raw and residual data. Thus, it appears that the shared variance between IS and RSMA actually dampened the IS signal at 15q21.1-q22.2. Finally, note in Figure 4 that 15q13.1-q14 and 15q21.1-22.2 both lie within a broader region in which we found evidence at genome-wide significance levels of linkage with ASD in our previous study with the same pedigrees [31]. Linkage evidence for ASD in the 15q13.1-q14 region is comparable with that for the two RSMA variables, but even stronger evidence of ASD linkage was observed in the 15q21.1-q22.2 region (Figure 4).

## Discussion

In a large sample of multiplex ASD pedigrees, we found evidence that IS and RMSA are distinct phenotypes that can be differentiated by both their phenotypic and genotypic relations. Further, the results suggest that ASD susceptibility loci vary in the breadth of their phenotypic effects. Finally, the results illustrate the value of using narrowly defined phenotypes to detect the specific contribution of implicated susceptibility loci to the heterogeneous ASD phenotype.

### IS and RSMA as distinct phenotypes

The overall pattern of relations of the two RRSB scales and their residuals with other ADI-R and ADOS measures suggests that although both RSMA and IS are indices of ASD severity, the relation with ASD severity is greater for RSMA than for IS and is in part a function of the shared variance between IS and RSMA. This general conclusion that RSMA is more closely associated with ASD severity is consistent with a previous report of the correlates of these scales [6]. The negative correlation between RSMA and IQ and the absence of a significant correlation between IS and IQ are consistent with previous reports [6,20], but the finding that the absolute magnitude of IQ correlations with both RSMA-Adj and IS-Adj is greater than IQ correlations with the raw scale values has not been reported previously. Taken together, these correlational findings suggest that the shared variance between IS and RSMA is associated with ASD severity but not with IQ.

The hypothesis that the positive relation between IS-Adj and IQ is mediated by anxiety is offered for further investigation. Anxiety, which is a common comorbid condition for ASD [60-62], has been reported to be positively correlated with IQ in children and adolescents with ASD [60,61]. If obsessive behaviors are attempts to regulate anxiety [63], then perhaps the positive relation between IS-Adj and IQ we observed is in part a consequence of the positive relation that others have reported between anxiety and IQ. Given that no data are available to support an association between the IS-Adj scale and anxiety, the hypothesis that the relation between IQ and IS-Adj is mediated by anxiety remains to be tested empirically.

Our results indicate that whereas both IS and RSMA are heritable, the estimated heritability was greater for IS. Further, the heritability of RSMA may not be independent of its relation with IS. Our findings are consistent with previous reports of significant heritability for IS [21,25], but in our families we find significantly positive heritability for RSMA as well. It is possible that the weaker RSMA heritability effect was not detected in those earlier reports.

Finally, we found different linkage patterns for IS and RSMA. There were many instances of suggestive signals linked to one but not the other phenotype, including differential linkage of IS at 9p24.3-p24.1 and 15q21.2-q22.2 (Table 2). The only signals that reached genome-wide significance were at different loci for each scale: 2q37.1-37.3 for IS and at 15q13.1-q14 for RSMA. It is true that at both 2q37.1-37.3 and 15q13.1-q14 there was suggestive evidence of linkage with the other scale (Table 3, Figure 3, Figure 4), but consideration of linkage results for residual scales and linkage results for ASD at the two loci suggests different interpretations of these suggestive signals. At 2q37.1-37.3, where there was a significant signal for IS, the suggestive signal for RSMA was not observed with RSMA-Adj and there was no linkage with ASD. Thus, it is possible that this region is relatively specific to IS, and that the suggestive signal for RSMA can be attributed to correlation of RSMA with IS. By contrast, at 15q13.1-q14, where there was a significant signal for RSMA, suggestive signals were found for both IS and IS-Adj, indicating that the IS signal was not due to the RSMA-IS correlation; the region was also linked to ASD in our earlier study. Thus, it seems likely that RSMA, being more strongly correlated with other ASD criteria, was more strongly linked to 15q13.1-q14, which appears to harbor risk markers for a broad range of ASD traits.

### Implications for studies of narrow phenotypes

Some of the IS- and RSMA-specific findings not replicated in our affected status analyses (for example, the significant signal specific to IS at 2q37.1-37.3) may be examples of the hoped-for outcome of identifying susceptibility loci that are specific to narrowly defined phenotypes [6]. Given that ASD is probably caused by many genes, each with relatively small effects [64,65], increasing our ability to detect such genes is crucial. Thus, these findings encourage further research with narrowly defined phenotypes to uncover linkage signals not observed with broader diagnostic categories.

Further, our findings provide an example of increased knowledge of the nature of genetic effects that may be possible with more homogeneous phenotypes. Previously, we reported possibly distinct ASD regions with evidence of linkage at 15q13.1-q14, 15q14-q21.1 and 15q21.1-q22.2 [31]. We now report that 15q13.1-q14 is linked to both RSMA and IS, but is linked more strongly to RSMA and that 15q21.1-q22.2 is linked to IS but not to RSMA. Thus, these two loci appear to affect different aspects of repetitive behavior, a possibility that was missed in our analysis of affected status.

The variability observed in this study in the phenotypic scope of linkage regions leads us to suggest that multiple ASD phenotypes should be used in future genetic studies to characterize the nature and breadth of the phenotypic linkage or association of risk variants. It is possible that variants with broad phenotypic effects may affect the root causes of ASD, whereas variants with narrow effects contribute to phenotypic heterogeneity among individuals with ASD. The use of multiple phenotypes emphasizes the importance of additional research aimed at developing an empirical model of the relations and interactions between specific features of ASD. Such a model should lead to identification of a set of phenotype measures that assess all the key specific features of ASD. The work of previous investigators to identify IS and RSMA as distinct features of repetitive behavior is a substantial contribution to this goal.

We note that our results are again consistent with the well-replicated finding of complexity and heterogeneity in ASD genetics. Our lod scores showed inter- and intra-family heterogeneity. For extended pedigrees, the scores expected under an assumption of a shared haplotype across all affected members exceeded by several lod units those actually found, depending upon the pedigree and model assumptions. Homogeneity clearly did not exist across all pedigrees in our sample; for any given region, multiple pedigrees showed no evidence of linkage.

### Previous genetic studies of repetitive and stereotyped behavior

Shao *et al*. [13] reported the only linkage study of which we are aware that stratified pedigrees on either IS or RSMA. That study differs from the present study in several regards. First, they limited their linkage analysis to the 15q11-q13 region, whereas we did a genome-wide scan. Second, they used nuclear families rather than extended pedigrees. Third, they used the diagnosis of AD as the phenotype, whereas we used IS and RSMA as phenotypes. Finally, they used ordered-subset analysis and we did not. Shao *et al*. did not find significant evidence of linkage in the 15q11-q13 region across all 81 families they studied but they did find significant evidence of linkage in the region of marker GABRB3 in the subset of 23 families with the highest mean IS scores. Stratifying families by RSMA or RRSB did not enhance the signal. GABRB3 is located at 24.4 Mb, which is upstream of the lower boundary (27.94 Mb) of 15q13.1-q14. We did not choose subsets of our sample, but rather re-defined affection status based on IS or RSMA phenotypic information, using information from all ASD members of the pedigrees. The methodological differences between our study and that of Shao *et al*. preclude firm conclusions about why they found that stratifying on IS but not RSMA enhanced the AD linkage signal, whereas we found both RSMA and IS, but particularly RSMA, to be associated with a region just downstream.

Studies that stratified pedigrees by other repetitive behavior measures, including individual RRSB items and the 'compulsions' factor examined by Tadevosyan-Lefer *et al*. [27], report increased HLOD scores for AD at chromosome 1 [7] and at 17q11.2 [10]. Significant associations between SLC25A12 alleles (2q31.1) have been reported for both the RRSB 'routines and rituals' category (similar to IS) [15] and the compulsions factor [16]. None of these loci overlaps signals that we obtained for IS or RSMA linkage. These differences may again be due in part to methodological differences between choosing subsets versus re-defining phenotypes.

The suggestive evidence of IS linkage that we observed on chromosome 9 for IS spans a region implicated as a susceptibility locus for OCD in two studies [66,67]. This replication is noteworthy because the earlier two studies did not include subjects with ASD. We did not find evidence of linkage for ASD diagnosis in this region using our full set of families, although we did find a evidence of linkage for ASD in this region in our analysis of a single large extended pedigree [68]. Previous research has indicated that OCD features in parents of children with AD are correlated with scores for IS but not RSMA in probands [30]. Thus, this region at the chromosome 9 telomere may underlie a repetitive behavior broader autism phenotype rather than ASD.

### Limitations

Our sample was a cohort of multiplex ASD pedigrees, and IS and RSMA data were collected only on subjects thought to have ASD. We believe our method is appropriate to the valid aim of uncovering susceptibility loci for ASD and related phenotypes within extended families containing multiple members with ASD. However, we acknowledge that our method limits the generalizability of our findings to other research aims. For example, the absence of repetitive behavior phenotype data for family members without ASD limits our ability to answer the question of whether repetitive behavior is a broader autism phenotype that occurs in unaffected relatives [30,69]. Further, because our sample is not population-based, we cannot generalize our findings to the search for genetic markers for repetitive behavior in the general population [3]. Finally, our study includes analyses of the IS and RSMA phenotypes under two simple dominant and recessive models. If we conservatively assume that these models and phenotypes are not correlated, then significance thresholds would be adjusted by log_10_(4) = 0.6 lod score units. Our thresholds would then be 2.26 for suggestive evidence and 3.9 for significant evidence. With this adjustment, results on chromosome 15 remain significant and many other results remain suggestive, but other results would be considered as nominal.

## Conclusions

IS and RSMA, two factors within the ADI-RRSB domain, were found to be linked to largely non-overlapping chromosomal regions. Genome-wide significance was observed for IS at 2q37.1-q37.3 (dominant model HLOD = 3.42) and for RSMA at 15q13.1-q14 (recessive model HLOD = 3.93). Regions varied in the range of phenotypes with which they were linked. These findings support the value of including multiple, narrowly defined phenotypes in ASD genetic research.

## Competing interests

HC, WMM and JMM received partial salary support from Lineagen Inc. http://www.lineagen.com from 12/1/07 to 12/31/08. This salary support is not ongoing.

## Authors' contributions

DSC conducted the statistical analyses and drafted the manuscript. JMM confirmed research diagnoses of ASD, supervised collection of all phenotype data and made significant contributions to the interpretation of results. RJR and KAB assisted with interpretation of results and helped draft the manuscript. MEV and NKW verified the accuracy of data extracted from a research database and contributed to the interpretation of results. WMM participated in the design of the study and helped draft the manuscript. HC conceived of the study, participated in its design, directed the statistical analyses and helped to draft the manuscript. All authors read and approved the final manuscript.
